# Supplementary material for: Analysis of Flavonoid Metabolites in Citrus Peels (Citrus reticulata “Dahongpao”) Using UPLC-ESI-MS/MS
Source: Molecules. 2019 Jul 24;24(15):2680. doi: 10.3390/molecules24152680 (PMC6696472; doi:10.3390/molecules24152680)
Supplement: Supplementary file 1 [file molecules-24-02680-s001.zip › Supplementary/Supplementary Table 1.docx]

Supplementary Table 1 A list of the 254 metabolites detected in this study.

| all_ID | Q1 (Da) | Q3 (Da) | Rt (min) | Molecular Weight (Da) | Ionization model | KEGG ID | Compounds |
| --- | --- | --- | --- | --- | --- | --- | --- |
| pma0249 | 479.1 | 317.1 | 3.52 | 478.12 | [M+H]+ | - | Selgin 5-O-hexoside |
| pma0253 | 477.1 | 315 | 5.27 | 476.14 | [M+H]+ | - | O-methylChrysoeriol 5-O-hexoside |
| pma0724 | 435.1 | 285.1 | 3.79 | 434.1 | [M+H]+ | - | Naringenin C-hexoside |
| pma0760 | 565 | 317 | 4.33 | 564 | [M+H]+ | - | Selgin O-malonylhexoside |
| pma0791 | 521 | 273 | 4.5 | 520 | [M+H]+ | - | Naringenin O-malonylhexoside |
| pma0795 | 551 | 303.1 | 4.56 | 550 | [M+H]+ | - | Tricetin O-malonylhexoside |
| pma0825 | 503 | 255 | 5.24 | 502 | [M+H]+ | - | Chrysin O-malonylhexoside |
| pma1087 | 565.2 | 499.2 | 3.77 | 564.16 | [M+H]+ | - | C-pentosyl-C-hexosyl-apigenin |
| pma1108 | 433.1 | 283.1 | 3.78 | 432.11 | [M+H]+ | - | Apigenin C-glucoside |
| pma1116 | 301.1 | 286 | 4.13 | 300.1 | [M+H]+ | C10098 | Kaempferide |
| pma1590 | 463.1 | 301 | 2.89 | 463.12 | [M]+ | - | Peonidin O-hexoside |
| pma2088 | 627.1 | 465 | 4.83 | 626.1 | [M+H]+ | - | Quercetin-3,4'-O-di-beta-glucopyranoside |
| pma6218 | 419.1 | 383.1 | 3.84 | 418.1 | [M+H]+ | - | O-methylnaringenin C-pentoside |
| pma6353 | 417.2 | 297.1 | 4 | 416.2 | [M+H]+ | - | Chrysin C-hexoside |
| pma6371 | 611.2 | 473.1 | 3.08 | 610.2 | [M+H]+ | - | di-C,C-hexosyl-luteolin |
| pma6373 | 671.1 | 347 | 4.06 | 670.1 | [M+H]+ | - | 3',4',5'-Dihydrotricetin O-hexosyl-O-hexoside |
| pma6496 | 449.1 | 299.1 | 3.45 | 448.1 | [M+H]+ | - | Luteolin 6-C-glucoside |
| pma6499 | 509.3 | 347.1 | 3.41 | 508.3 | [M+H]+ | - | Limocitrin O-hexoside |
| pma6516 | 757.3 | 595.2 | 3.55 | 756.3 | [M+H]+ | - | C-hexosyl-apigenin O-hexosyl-O-hexoside |
| pma6586 | 567.3 | 405.2 | 4.04 | 566.3 | [M+H]+ | - | Tricin O-glycerylhexoside |
| pma6639 | 479.2 | 317.2 | 4.17 | 478.2 | [M+H]+ | - | Isorhamnetin O-hexoside |
| pma6647 | 625.2 | 607.1 | 3.37 | 624.2 | [M+H]+ | - | C-hexosyl-chrysoeriol O-hexoside |
| pmb0322 | 653.3 | 344.9 | 4.76 | 652.3 | [M+H]+ | - | 3’,4’,5’-Tricetin O-rutinoside |
| pmb0358 | 641.1 | 479.1 | 4.39 | 640.1 | [M+H]+ | - | Selgin O-hexosyl-O-hexoside |
| pmb0541 | 697.1 | 534.9 | 2.09 | 697.1 | [M]+ | - | Cyanidin 3-O-glucosyl-malonylglucoside |
| pmb0558 | 637.1 | 303.4 | 3.27 | 637.1 | [M]+ | - | Delphinidin O-malonyl-malonylhexoside |
| pmb0565 | 509.1 | 347.2 | 3.41 | 508.1 | [M+H]+ | - | Syringetin 3-O-hexoside |
| pmb0566 | 581.2 | 383.2 | 3.74 | 580.2 | [M+H]+ | - | Luteolin O-hexosyl-O-pentoside |
| pmb0569 | 509.2 | 347.1 | 4.17 | 508.2 | [M+H]+ | - | Syringetin 5-O-hexoside |
| pmb0576 | 519 | 271 | 4.23 | 518 | [M+H]+ | - | Apigenin O-malonylhexoside |
| pmb0579 | 669 | 207 | 4.92 | 668 | [M+H]+ | - | Chrysoeriol O-sinapoylhexoside |
| pmb0587 | 639.1 | 477 | 3.29 | 638.1 | [M+H]+ | - | Chrysoeriol O-glucuronic acid-O-hexoside |
| pmb0588 | 611.1 | 449.3 | 3.32 | 610.1 | [M+H]+ | - | Luteolin 3',7-di-O-glucoside |
| pmb0595 | 479.2 | 317.2 | 4.17 | 478.2 | [M+H]+ | - | Isorhamnetin 5-O-hexoside |
| pmb0602 | 509.3 | 347.2 | 4.17 | 508.3 | [M+H]+ | - | Syringetin 7-O-hexoside |
| pmb0604 | 449.1 | 287.6 | 3.93 | 448.1 | [M+H]+ | C12249 | Kaempferol 3-O-glucoside (Astragalin) |
| pmb0613 | 757.1 | 577.2 | 2.68 | 756.1 | [M+H]+ | - | Apigenin 6-C-hexosyl-8-C-hexosyl-O-hexoside |
| pmb0618 | 627.1 | 465.1 | 2.78 | 626.1 | [M+H]+ | - | 8-C-hexosyl-hesperetin O-hexoside |
| pmb0622 | 611.2 | 473.1 | 3.09 | 610.2 | [M+H]+ | - | C-hexosyl-luteolin O-hexoside |
| pmb0623 | 625.2 | 607.1 | 2.96 | 624.2 | [M+H]+ | - | 6-C-hexosyl chrysoeriol O-hexoside |
| pmb0628 | 613.1 | 451.1 | 3.09 | 612.1 | [M+H]+ | - | Eriodictiol C-hexosyl-O-hexoside |
| pmb0639 | 757.2 | 595.2 | 3.13 | 756.2 | [M+H]+ | - | 8-C-hexosyl-apigenin O-hexosyl-O-hexoside |
| pmb0645 | 627.1 | 465.2 | 3.41 | 626.1 | [M+H]+ | - | 6-C-hexosyl-hesperetin O-hexoside |
| pmb0652 | 565.1 | 433.3 | 3.6 | 564.1 | [M+H]+ | - | C-hexosyl-apigenin O-pentoside |
| pmb0653 | 595.1 | 463.1 | 3.68 | 594.1 | [M+H]+ | - | di-C,C-hexosyl-apigenin |
| pmb0661 | 609.1 | 463.3 | 3.87 | 608.1 | [M+H]+ | - | Chrysoeriol C-hexosyl-O-rhamnoside |
| pmb0665 | 611.2 | 465.1 | 3.78 | 610.2 | [M+H]+ | - | Luteolin 8-C-hexosyl-O-hexoside |
| pmb0682 | 801 | 301.2 | 4.31 | 800 | [M+H]+ | - | 8-C-hexosyl-apigenin O-sinapoylhexoside |
| pmb0686 | 537 | 289 | 4.41 | 536 | [M+H]+ | - | Eriodictyol O-malonylhexoside |
| pmb0691 | 757.1 | 431.4 | 3.09 | 756.1 | [M+H]+ | - | Luteolin C-hexosyl-O-rhamnoside O-hexoside |
| pmb0693 | 565.1 | 433.3 | 3.39 | 564.1 | [M+H]+ | - | C-hexosyl-apigenin C-pentoside |
| pmb0696 | 625 | 463 | 3.46 | 624 | [M+H]+ | - | 8-C-hexosyl chrysoeriol O-hexoside |
| pmb0706 | 713.1 | 465.1 | 2.96 | 712.1 | [M+H]+ | - | Quercetin 5-O-malonylhexosyl-hexoside |
| pmb0711 | 611.2 | 303.1 | 3.7 | 610.2 | [M+H]+ | - | Quercetin 7-O-rutinoside |
| pmb0712 | 655.2 | 331.1 | 3.5 | 654.2 | [M+H]+ | - | Tricin 5-O-hexosyl-O-hexoside |
| pmb0713 | 655.2 | 331.2 | 3.37 | 654.2 | [M+H]+ | - | Tricin 7-O-hexosyl-O-hexoside |
| pmb0720 | 579.1 | 331.1 | 4.56 | 578.1 | [M+H]+ | - | Tricin O-malonylhexoside |
| pmb0736 | 493.1 | 331 | 4.3 | 492.1 | [M+H]+ | - | Tricin 7-O-hexoside |
| pmb0739 | 659.3 | 331 | 4.92 | 658.3 | [M+H]+ | - | Tricin O-hexosyl-O-syringin alcohol |
| pmb0835 | 611.2 | 287.1 | 2.36 | 610.2 | [M+H]+ | - | Gallocatechin-gallocatechin |
| pmb1312 | 689.2 | 331.2 | 4.43 | 688.2 | [M+H]+ | - | Tricin 4'-O-(β-guaiacylglyceryl) ether 7-O-hexoside |
| pmb1466 | 511.2 | 331.1 | 6.38 | 510.2 | [M+H]+ | - | Tricin 4'-O-syringic acid |
| pmb2586 | 593.1 | 285.6 | 3.72 | 594.1 | [M-H]- | - | Gallocatechin-catechin |
| pmb2831 | 315.1 | 153.1 | 2.44 | 316.1 | [M-H]- | - | Protocatechuic acid O-glucoside |
| pmb2850 | 329 | 314 | 5.73 | 330 | [M-H]- | - | Tricin |
| pmb2954 | 771.1 | 609.3 | 2.83 | 772.1 | [M-H]- | - | Luteolin O-hexosyl-O-hexosyl-O-hexoside |
| pmb2957 | 465.1 | 285.1 | 2.59 | 466.1 | [M-H]- | - | Cyanidin O-syringic acid |
| pmb2979 | 549.2 | 387.1 | 3.91 | 550.2 | [M-H]- | - | Hesperetin O-malonylhexoside |
| pmb2984 | 491.1 | 287.1 | 5.09 | 492.1 | [M-H]- | - | Acetyl-eriodictyol O-hexoside |
| pmb2987 | 487.1 | 163 | 2.63 | 488.1 | [M-H]- | - | Acacetin O-acetyl hexoside |
| pmb3002 | 607.1 | 299 | 3.98 | 608.1 | [M-H]- | - | Chrysoeriol 7-O-rutinoside |
| pmb3013 | 519.1 | 314.2 | 4.32 | 520.1 | [M-H]- | - | Isorhamnetin O-acetyl-hexoside |
| pmb3023 | 449.1 | 287 | 3.37 | 450.1 | [M-H]- | - | Eriodictyol C-hexoside |
| pmb3024 | 447.1 | 327.1 | 3.45 | 448.1 | [M-H]- | - | Luteolin C-hexoside |
| pmb3026 | 505.1 | 301.2 | 3.8 | 506.1 | [M-H]- | - | Quercetin O-acetylhexoside |
| pmb3028 | 535 | 491.3 | 3.75 | 536 | [M-H]- | - | Tricin O-sinapic acid |
| pmb3041 | 521.1 | 329.2 | 3.81 | 522.1 | [M-H]- | - | Tricin O-saccharic acid |
| pmb3053 | 523.1 | 329.2 | 6 | 524.1 | [M-H]- | - | Tricin O-eudesmic acid |
| pmb3894 | 329.1 | 229.1 | 5.91 | 330.1 | [M-H]- | - | Di-O-methylquercetin |
| pmc1990 | 299.1 | 223.1 | 6.78 | 300.1 | [M-H]- | - | 4'-Hydroxy-5,7-dimethoxyflavanone |
| pme0001 | 609.2 | 301 | 4.07 | 610.19 | [M-H]- | C09806 | Hesperetin 7-O-neohesperidoside (Neohesperidin) |
| pme0196 | 285 | 151 | 5.73 | 286.05 | [M-H]- | C05903 | Kaempferol |
| pme0199 | 301 | 151 | 5.12 | 302.04 | [M-H]- | C00389 | Quercetin |
| pme0202 | 611.2 | 303 | 3.7 | 610.15 | [M+H]+ | C05625 | Quercetin 3-O-rutinoside (Rutin) |
| pme0321 | 431.1 | 285 | 4.94 | 432.11 | [M-H]- | - | Kaempferol 7-O-rhamnoside |
| pme0333 | 579.2 | 271 | 4.01 | 578.16 | [M+H]+ | C12627 | Apigenin 7-O-neohesperidoside (Rhoifolin) |
| pme0359 | 433.1 | 271 | 3.81 | 432.11 | [M+H]+ | - | Apigenin 5-O-glucoside |
| pme0364 | 301.1 | 286 | 5.77 | 300.06 | [M+H]+ | C04293 | Chrysoeriol |
| pme0372 | 435.1 | 273 | 4.22 | 434.12 | [M+H]+ | C09099 | Naringenin 7-O-glucoside (Prunin) |
| pme0374 | 431.1 | 311 | 3.79 | 432.11 | [M-H]- | C01714 | Isovitexin |
| pme0377 | 273.1 | 153 | 5.59 | 272.07 | [M+H]+ | C00509 | Naringenin |
| pme0379 | 271.1 | 153 | 5.63 | 270.05 | [M+H]+ | C01477 | Apigenin |
| pme0442 | 303 | 229 | 2.98 | 303.24 | [M]+ | C05908 | Delphinidin |
| pme0443 | 493 | 331 | 2.94 | 493 | [M]+ | - | Malvidin 3-O-galactoside |
| pme0444 | 493.2 | 331 | 2.92 | 493.2 | [M]+ | C12140 | Malvidin 3-O-glucoside (Oenin) |
| pme1201 | 273.1 | 167 | 5.56 | 274.08 | [M-H]- | C00774 | Phloretin |
| pme1398 | 465.1 | 303 | 2.26 | 465.1 | [M]+ | C12138 | Delphinidin 3-O-glucoside (Mirtillin) |
| pme1399 | 355.2 | 178.5 | 8.4 | 354.15 | [M+H]+ | C16417 | Xanthohumol |
| pme1478 | 317 | 151 | 4.7 | 318.04 | [M-H]- | C10107 | Myricetin |
| pme1486 | 457.2 | 169 | 3.33 | 458.09 | [M-H]- | C09731 | Epigallate catechin gallate (EGCG) |
| pme1518 | 403.1 | 373 | 7.06 | 402.13 | [M+H]+ | C10112 | Nobiletin |
| pme1521 | 303.1 | 125 | 4.15 | 304.06 | [M-H]- | C01617 | Dihydroquercetin (Taxifolin) |
| pme1535 | 305 | 125 | 2.28 | 306.07 | [M-H]- | C12127 | (+)-Gallocatechin (GC) |
| pme1540 | 625.2 | 317 | 3.66 | 624.17 | [M+H]+ | - | Isorhamnetin 3-O-neohesperidoside |
| pme1550 | 373.1 | 343 | 7.54 | 372.12 | [M+H]+ | C10190 | Tangeretin |
| pme1562 | 441.3 | 169 | 3.89 | 442.3 | [M-H]- | - | Epicatechin gallate (ECG) |
| pme1580 | 287.1 | 135 | 5.05 | 288.06 | [M-H]- | C05631 | Eriodictyol |
| pme1587 | 417.1 | 255 | 3.36 | 416.11 | [M+H]+ | C10216 | Daidzein 7-O-glucoside (Daidzin) |
| pme1590 | 317.1 | 153 | 5.87 | 316.06 | [M+H]+ | C10084 | Isorhamnetin |
| pme1598 | 463.1 | 301 | 3.85 | 464.13 | [M-H]- | - | Hesperetin 5-O-glucoside |
| pme1665 | 593.2 | 311 | 3.36 | 594.16 | [M-H]- | C08064 | Isovitexin 7-O-glucoside (Saponarin) |
| pme1773 | 595 | 287 | 2.7 | 595 | [M]+ | C08620 | Cyanidin 3-O-rutinoside (Keracyanin) |
| pme1777 | 611 | 287 | 2.08 | 611 | [M]+ | C08639 | Cyanidin 3,5-O-diglucoside (Cyanin) |
| pme1824 | 153.1 | 109 | 2.48 | 154.03 | [M-H]- | C00230 | Protocatechuic acid |
| pme2247 | 303 | 285 | 6.57 | 302.01 | [M+H]+ | C10788 | Ellagic acid |
| pme2293 | 367.1 | 149 | 7.38 | 368.13 | [M-H]- | C10443 | Curcumin |
| pme2319 | 301.1 | 164 | 5.75 | 302.08 | [M-H]- | C01709 | Hesperetin |
| pme2459 | 449.1 | 287.2 | 3.87 | 448.1 | [M+H]+ | C03951 | Luteolin 7-O-glucoside (Cynaroside) |
| pme2478 | 137.1 | 108.4 | 3.06 | 138.03 | [M-H]- | C16700 | Protocatechuic aldehyde |
| pme2898 | 321.1 | 303 | 3.52 | 320.05 | [M+H]+ | C02906 | Dihydromyricetin |
| pme2949 | 609.2 | 301 | 4.08 | 610.19 | [M-H]- | C09755 | Hesperetin 7-rutinoside (Hesperidin) |
| pme2957 | 271.1 | 151 | 5.57 | 272.07 | [M-H]- | C06561 | Naringenin chalcone |
| pme2963 | 287.1 | 259 | 4.62 | 288.06 | [M-H]- | C00974 | Aromadedrin (Dihydrokaempferol) |
| pme2975 | 677.2 | 531 | 4.98 | 676.24 | [M+H]+ | C17555 | Icariin (kaempferol 3,7-O-diglucoside 8-prenyl derivative) |
| pme2984 | 593 | 285 | 5.09 | 594.2 | [M-H]- | C09830 | Isosakuranetin-7-neohesperidoside (Poncirin) |
| pme3129 | 463 | 301 | 3.86 | 464.1 | [M-H]- | - | Quercetin 4'-O-glucoside (Spiraeoside) |
| pme3134 | 237 | 209 | 7.73 | 238.06 | [M-H]- | C20871 | 3-Hydroxyflavone |
| pme3208 | 445 | 283 | 3.54 | 446.12 | [M-H]- | C16195 | Glycitin |
| pme3209 | 431 | 269 | 4.01 | 432.11 | [M-H]- | C09126 | Genistein 7-O-Glucoside (Genistin) |
| pme3224 | 577 | 413 | 3.67 | 578.16 | [M-H]- | C12628 | Vitexin 2''-O-beta-L-rhamnoside |
| pme3256 | 611 | 303 | 2.37 | 611 | [M]+ | C16315 | Delphinidin 3-O-rutinoside (Tulipanin) |
| pme3268 | 449 | 287 | 3.87 | 448.1 | [M+H]+ | C12626 | Kaempferol 3-O-galactoside (Trifolin) |
| pme3279 | 287 | 153 | 4.89 | 286.05 | [M+H]+ | C12134 | 2'-Hydroxygenistein |
| pme3282 | 273 | 229 | 3.4 | 274.08 | [M-H]- | C09320 | Afzelechin (3,5,7,4'-Tetrahydroxyflavan) |
| pme3290 | 331 | 316 | 6.63 | 330.07 | [M+H]+ | C01265 | 3,7-Di-O-methylquercetin |
| pme3297 | 433 | 287 | 4.49 | 432.11 | [M+H]+ | C16911 | Kaempferol 3-O-rhamnoside (Kaempferin) |
| pme3300 | 301 | 151 | 4.51 | 302.04 | [M-H]- | C10192 | Tricetin |
| pme3392 | 433.1 | 271 | 2.83 | 433.1 | [M]+ | - | Pelargonidin 3-O-beta-D-glucoside（Callistephin chloride) |
| pme3401 | 345 | 330 | 5.78 | 346.07 | [M-H]- | C11620 | Syringetin |
| pme3407 | 331 | 151 | 5.15 | 332.05 | [M-H]- | C12633 | Laricitrin |
| pme3461 | 301.1 | 151 | 5.75 | 302.28 | [M-H]- | C09756 | Homoeriodictyol |
| pme3464 | 285.1 | 164 | 6.98 | 286.08 | [M-H]- | C05334 | Isosakuranetin (4'-Methylnaringenin) |
| pme3469 | 741.2 | 433 | 3.35 | 740.22 | [M+H]+ | C10178 | Kaempferol-3-O-robinoside-7-O-rhamnoside (Robinin) |
| pme3473 | 271.1 | 151 | 5.59 | 272.07 | [M-H]- | C09614 | Butin |
| pme3502 | 429.1 | 267 | 4.59 | 430.13 | [M-H]- | C10509 | Formononetin 7-O-glucoside (Ononin) |
| pme3514 | 301 | 151 | 5.11 | 302.04 | [M-H]- | C10105 | Morin |
| pme3609 | 287 | 213 | 3.54 | 287.24 | [M]+ | C05905 | Cyanidin |
| pmf0011 | 593.2 | 473 | 3.19 | 594.16 | [M-H]- | - | Apigenin 6,8-C-diglucoside |
| pmf0012 | 595.2 | 457.2 | 3.18 | 594.16 | [M+H]+ | - | 6,8-di-C-glucoside Apigenine |
| pmf0027 | 449.1 | 286.8 | 2.68 | 448.1 | [M+H]+ | C08647 | Cyanidin 3-O-galactoside |
| pmf0057 | 271.1 | 151 | 5.66 | 272.07 | [M-H]- | C06561 | 4,2',4',6'-Tetrahydroxychalcone |
| pmf0058 | 271 | 150.9 | 5.66 | 272.07 | [M-H]- | C00509 | 4',5,7-Trihydroxyflavanone |
| pmf0116 | 627.2 | 303.1 | 1.88 | 662.12 | [M-Cl]+ | C16312 | Delphin chloride |
| pmf0127 | 405.1 | 243.1 | 5.09 | 404.15 | [M+H]+ | - | Deoxyrhapontin |
| pmf0203 | 463.1 | 301.1 | 2.94 | 498.09 | [M-Cl]+ | - | Peonidin 3-O-glucoside chloride |
| pmf0204 | 465.1 | 303 | 3.73 | 464.1 | [M+H]+ | - | Hyperoside |
| pmf0232 | 593.1 | 285 | 4.92 | 594.14 | [M-H]- | C17140 | Tiliroside |
| pmf0234 | 563.1 | 443.1 | 3.53 | 564.15 | [M-H]- | - | Isoschaftoside |
| pmf0247 | 449.1 | 299.2 | 3.46 | 448.1 | [M+H]+ | C10114 | Orientin |
| pmf0260 | 373.1 | 312.8 | 6.58 | 372.12 | [M+H]+ | C10186 | Sinensetin |
| pmf0277 | 519.2 | 501.2 | 10.75 | 518.19 | [M+H]+ | C07667 | Gossypol |
| pmf0278 | 465.1 | 300.7 | 4 | 464.1 | [M-H]- | - | Gossypitrin |
| pmf0360 | 449.1 | 151 | 3.97 | 450.12 | [M-H]- | C17449 | Astilbin |
| pmf0363 | 301.1 | 258 | 6.28 | 300.06 | [M+H]+ | - | Hydroxygenkwanin |
| pmf0370 | 479.2 | 317 | 4.55 | 478.15 | [M+H]+ | - | Persicoside |
| pmf0371 | 315.1 | 300 | 4.72 | 316.06 | [M-H]- | C10119 | Pedalitin |
| pmf0375 | 479.1 | 317 | 4.18 | 478.11 | [M+H]+ | - | Isorhamnetin 3-O-glucoside |
| pmf0379 | 457.1 | 169 | 3.44 | 458.08 | [M-H]- | - | Gallocatechin gallate |
| pmf0381 | 563.1 | 353 | 3.5 | 564.15 | [M-H]- | - | Vicenin-3 |
| pmf0383 | 345.1 | 284 | 6.68 | 344.09 | [M+H]+ | C19807 | 5,7-Dihydroxy-3',4',5'-trimethoxyflavone |
| pmf0393 | 317.1 | 177 | 7.15 | 316.09 | [M+H]+ | - | Persicogenin |
| pmf0417 | 595.2 | 287 | 3.7 | 596.17 | [M-H]- | C09732 | Eriocitrin |
| pmf0453 | 339.1 | 177.1 | 7.31 | 338.12 | [M+H]+ | C17742 | Demethoxycurcumin |
| pmf0458 | 295.2 | 277 | 7.08 | 294.18 | [M+H]+ | C10462 | 6-Gingerol |
| pmf0492 | 415 | 267 | 3.12 | 416.11 | [M-H]- | C10524 | Puararin |
| pmf0548 | 563 | 353 | 3.34 | 596.14 | [M-H]- | C10181 | Schaftoside |
| pmf0552 | 579.1 | 403 | 8.75 | 580.14 | [M-H]- | C10182 | Sciadopitysin |
| pmf0567 | 299.1 | 211 | 5.72 | 300.06 | [M-H]- | C10534 | Tectorigenin |
| pmf0584 | 437.1 | 275 | 4.37 | 436.14 | [M+H]+ | C01604 | Phloridzin |
| pmf0617 | 787.23 | 463.4 | 2.23 | 787.23 | [M]+ | - | Peonidin 3-sophoroside-5-glucoside. |
| pmf0618 | 893.23 | 449.4 | 2.78 | 893.23 | [M]+ | - | Cyanidin 3-p-hydroxybenzoylsophoroside-5-glucoside |
| pma0214 | 479 | 317.1 | 3.91 | 478 | [M+H]+ | - | methylQuercetin O-hexoside |
| pma6558 | 313.1 | 298.1 | 7.22 | 314.08 | [M-H]- | - | Velutin |
| pma6576 | 347 | 153.2 | 4.41 | 346 | [M+H]+ | - | Spinacetin |
| pmb0545 | 477.1 | 315 | 3.32 | 477.1 | [M]+ | - | Rosinidin O-hexoside |
| pmb0557 | 621.1 | 287.3 | 3.26 | 621.1 | [M]+ | - | Cyanidin O-malonyl-malonylhexoside |
| pmb0563 | 301.1 | 286 | 3.95 | 301.1 | [M]+ | C08726 | Peonidin |
| pmb0578 | 655 | 207 | 4.67 | 654 | [M+H]+ | - | Luteolin O-sinapoylhexoside |
| pmb0603 | 625.1 | 301.6 | 4.17 | 624.1 | [M+H]+ | - | Chrysoeriol O-hexosyl-O-hexoside |
| pmb0605 | 433.1 | 271.1 | 4.21 | 432.1 | [M+H]+ | C04608 | Apigenin 7-O-glucoside (Cosmosiin) |
| pmb0608 | 549.1 | 301.4 | 4.55 | 548.1 | [M+H]+ | - | Chrysoeriol O-malonylhexoside |
| pmb0617 | 729.1 | 579 | 2.82 | 728.1 | [M+H]+ | - | C-hexosyl-apigenin O-hexosyl-O-pentoside |
| pmb0620 | 787.1 | 625.1 | 2.77 | 786.1 | [M+H]+ | - | Chrysoeriol 6-C-hexoside 8-C-hexoside-O-hexoside |
| pmb0621 | 641.1 | 622.8 | 3.09 | 640.1 | [M+H]+ | - | C-hexosyl-isorhamnetin O-hexoside |
| pmb0624 | 611.1 | 431.3 | 3.09 | 610.1 | [M+H]+ | - | 6-C-hexosyl-luteolin O-hexoside |
| pmb0626 | 757.1 | 433.4 | 3.1 | 756.1 | [M+H]+ | - | 6-C-hexosyl-apigenin O-hexosyl-O-hexoside |
| pmb0636 | 581.1 | 431.3 | 3.18 | 580.1 | [M+H]+ | - | 6-C-hexosyl luteolin O-pentoside |
| pmb0657 | 739.1 | 431.3 | 3.74 | 738.1 | [M+H]+ | - | C-rhamnosyl-acacetin O-p-coumaroylhexoside |
| pmb0666 | 801.1 | 639.1 | 3.82 | 800.1 | [M+H]+ | - | 6-C-hexosyl-apigenin O-sinapoylhexoside |
| pmb0667 | 757.1 | 177.1 | 3.87 | 756.1 | [M+H]+ | - | C-hexosyl-luteolin O-feruloylpentoside |
| pmb0678 | 771.2 | 177.1 | 4.26 | 770.2 | [M+H]+ | - | 8-C-hexosyl-apigenin O-feruloylhexoside |
| pmb0689 | 463.1 | 331.2 | 4.75 | 462.1 | [M+H]+ | - | Chrysoeriol C-hexoside |
| pmb0695 | 771.1 | 606.8 | 3.44 | 770.1 | [M+H]+ | - | C-hexosyl-chrysoeriol O-rutinoside |
| pmb0701 | 463.1 | 313.2 | 3.89 | 462.1 | [M+H]+ | - | Chrysoeriol 8-C-hexoside |
| pmb0719 | 659.2 | 331.1 | 4.5 | 658.2 | [M+H]+ | - | Tricin 4'-O-(syringyl alcohol) ether 5-O-hexoside |
| pmb0725 | 669.1 | 331.4 | 5.1 | 668.1 | [M+H]+ | - | Tricin 7-O-feruloylhexoside |
| pmb0733 | 639.1 | 331.4 | 4.07 | 638.1 | [M+H]+ | - | Tricin 5-O-rutinoside |
| pmb0738 | 699.2 | 331.1 | 4.88 | 698.2 | [M+H]+ | - | Tricin O-sinapoylhexoside |
| pmb1108 | 773.1 | 593 | 2.66 | 772.1 | [M+H]+ | - | Luteolin 6-C-hexoside 8-C-hexosyl-O-hexoside |
| pmb2973 | 740.1 | 431.4 | 3.34 | 741.1 | [M-H]- | - | Chrysoeriol 6-C-pentosyl-O-rutinoside |
| pmb2997 | 799.1 | 461.1 | 3.96 | 800.1 | [M-H]- | - | Chrysoeriol O-hexosyl-O-hexosyl-O-Glucuronic acid |
| pmb3000 | 503.1 | 341.1 | 3.94 | 504.1 | [M-H]- | - | Chrysoeriol O-acetylhexoside |
| pmb3012 | 461.1 | 299.2 | 4.32 | 462.1 | [M-H]- | - | Chrysoeriol 7-O-hexoside |
| pmb3042 | 491.1 | 329.1 | 4 | 492.1 | [M-H]- | - | Tricin 5-O-hexoside |
| pmb3044 | 653.1 | 329.2 | 4.16 | 654.1 | [M-H]- | - | Tricin di-O-hexoside |
| pme0088 | 285 | 151 | 5 | 286 | [M-H]- | C01514 | Luteolin |
| pme0330 | 579.2 | 271 | 4.17 | 580.18 | [M-H]- | C09789 | Naringenin 7-O-neohesperidoside (Naringin) |
| pme0361 | 435.1 | 303 | 3.98 | 434.08 | [M+H]+ | - | Quercetin 3-alpha-L-arabinofuranoside (Avicularin) |
| pme0367 | 577.2 | 269 | 4.01 | 578.16 | [M-H]- | - | Apigenin 7-rutinoside (Isorhoifolin) |
| pme0369 | 593.2 | 285 | 3.83 | 594.16 | [M-H]- | - | Kaempferol 3-O-rutinoside (Nicotiflorin) |
| pme1500 | 313.1 | 283 | 7.23 | 314.08 | [M-H]- | - | Kumatakenin |
| pme1541 | 283.1 | 268 | 7.06 | 284.07 | [M-H]- | C01470 | Acacetin |
| pme1568 | 285 | 257 | 5.08 | 286.05 | [M-H]- | C10510 | Orobol (5,7,3',4'-tetrahydroxyisoflavone) |
| pme1599 | 301.1 | 165 | 6.28 | 302.08 | [M-H]- | - | 7-O-Methyleriodictyol |
| pme1605 | 593.2 | 285 | 3.76 | 594.16 | [M-H]- | - | Kaempferol 3-O-robinobioside (Biorobin) |
| pme1793 | 595 | 270.9 | 2.38 | 595 | [M]+ | C08725 | Pelargonin |
| pme2977 | 347.3 | 285 | 6.66 | 346.25 | [M+H]+ | - | Troxerutin (Trihydroxyethyl rutin) |
| pme3212 | 465 | 303 | 3.87 | 464.1 | [M+H]+ | C05623 | Quercetin 3-O-glucoside (Isotrifoliin) |
| pme3250 | 285 | 270 | 7 | 284.07 | [M+H]+ | C00814 | Biochanin A |
| pme3292 | 283 | 268 | 6.97 | 284.07 | [M-H]- | C10521 | Prunetin |
| pmf0005 | 579.2 | 271 | 4.05 | 580.53 | [M-H]- | C09793 | Narirutin |
| pmf0179 | 623.2 | 315.1 | 4.04 | 624.17 | [M-H]- | - | Narcissoside |
| pmf0208 | 465.1 | 303 | 3.9 | 464.1 | [M+H]+ | - | Isoquercitroside |
| pmf0236 | 563.1 | 241.2 | 4.68 | 564.13 | [M-H]- | - | Theaflavin |
| pmf0549 | 607.2 | 299 | 4.14 | 608.17 | [M-H]- | C10039 | Diosmin |
| pmf0614 | 625.4 | 301 | 2 | 625.4 | [M]+ | - | Peonidin 3, 5-diglucoside chloride |
| pma6518 | 771.2 | 177.1 | 4.25 | 770.21 | [M+H]+ | - | C-pentosyl-chrysoeriol 7-O-feruloylhexoside |
| pmb0601 | 595.1 | 301.4 | 4.12 | 594.1 | [M+H]+ | - | Chrysoeriol O-hexosyl-O-pentoside |
| pmb0676 | 801 | 207.1 | 4.18 | 800 | [M+H]+ | - | 8-C-hexosyl-chrysoeriol O-feruloylhexoside |
| pmb2969 | 625.1 | 445.2 | 2.47 | 626.1 | [M-H]- | - | Hesperetin C-hexoside O-hexoside |
| pmb2978 | 563.1 | 269.1 | 4.04 | 564.1 | [M-H]- | - | Apigenin O-hexosyl-O-pentoside |
| pmb2991 | 739.1 | 269.2 | 3.79 | 740.1 | [M-H]- | - | Apigenin O-hexosyl-O-rutinoside |
| pmb2999 | 461.1 | 299.1 | 3.87 | 462.1 | [M-H]- | - | Chrysoeriol 5-O-hexoside |
| pme3369 | 315 | 165 | 6.43 | 316.06 | [M-H]- | C10176 | Rhamnetin (7-O-methxyl quercetin) |
| pmf0472 | 565.2 | 271.1 | 4.06 | 564.15 | [M+H]+ | C04858 | Apiin |
| pmf0551 | 269.1 | 226 | 8.57 | 268.07 | [M+H]+ | C11621 | Tectochrysin |
| pmf0616 | 697.1 | 535.1 | 3.03 | 697.1 | [M]+ | - | Malvidin 3-acetyl-5-diglucoside |
| pma6638 | 477.2 | 315 | 5.3 | 476.2 | [M+H]+ | - | O-methylChrysoeriol 7-O-hexoside |
| pmb0648 | 479.1 | 383.5 | 3.53 | 478.1 | [M+H]+ | - | Isorhamnetin C-hexoside |
| pmb0745 | 497.1 | 331.3 | 5.75 | 496.1 | [M+H]+ | - | Tricin 4'-O-syringyl alcohol |
| pmb0746 | 527.1 | 331 | 5.84 | 526.1 | [M+H]+ | - | Tricin 4'-O-β-guaiacylglycerol |
| pmb3032 | 561.1 | 329.1 | 5.69 | 562.1 | [M-H]- | - | Tricin O-malonyl rhamnoside |
| pmb3049 | 657.1 | 329.2 | 4.72 | 658.1 | [M-H]- | - | Tricin 4'-O-(syringyl alcohol) ether 7-O-hexoside |
| pma0779 | 725.2 | 331.1 | 4.5 | 724.2 | [M+H]+ | - | Tricin O-rhamnosyl-O-malonylhexoside |
