# Supplementary figures and images for: Analysis of Flavonoid Metabolites in Citrus Peels (Citrus reticulata “Dahongpao”) Using UPLC-ESI-MS/MS
Source: Molecules. 2019 Jul 24;24(15):2680. doi: 10.3390/molecules24152680 (PMC6696472; doi:10.3390/molecules24152680)

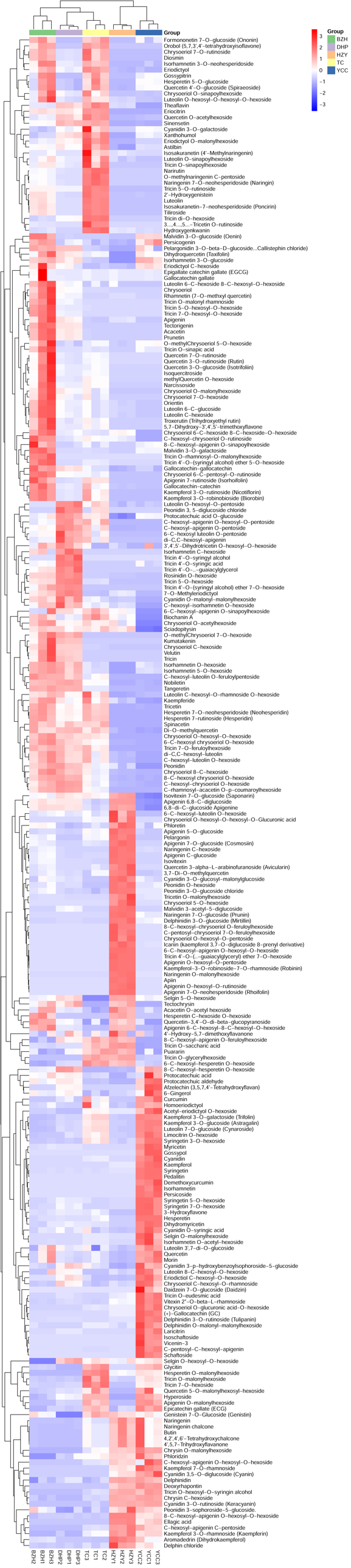

Supplement: Supplementary file 1 [file molecules-24-02680-s001.zip › Supplementary/Supplementary Fig 1.jpg]
