# Supplementary material for: Analysis of Flavonoid Metabolites in Citrus Peels (Citrus reticulata “Dahongpao”) Using UPLC-ESI-MS/MS
Source: Molecules. 2019 Jul 24;24(15):2680. doi: 10.3390/molecules24152680 (PMC6696472; doi:10.3390/molecules24152680)
Supplement: Supplementary file 1 [file molecules-24-02680-s001.zip › Supplementary/Supplementary Table 2.doc]

Supplementary Table 2 Up-regulation and down-regulation data of compounds in each Comparison group

| group name | All sig diff | down-regulated | up-regulated |
| --- | --- | --- | --- |
| BZH_vs_HZY | 98 | 67 | 31 |
| BZH_vs_TC | 64 | 40 | 24 |
| DHP_vs_BZH | 52 | 21 | 31 |
| DHP_vs_HZY | 100 | 64 | 36 |
| DHP_vs_TC | 68 | 41 | 27 |
| DHP_vs_YCC | 97 | 65 | 32 |
| HZY_vs_TC | 97 | 35 | 62 |
| YCC_vs_BZH | 98 | 31 | 67 |
| YCC_vs_HZY | 71 | 32 | 39 |
| YCC_vs_TC | 98 | 34 | 64 |
